# Recent Progress in Modeling and Simulation of Biomolecular Crowding and Condensation Inside Cells

**DOI:** 10.1021/acs.jcim.4c01520

**Published:** 2024-12-11

**Authors:** Apoorva Mathur, Rikhia Ghosh, Ariane Nunes-Alves

**Affiliations:** † Institute of Chemistry, Technische Universität Berlin, Straße des 17. Juni 135, 10623 Berlin, Germany; ‡ Boehringer Ingelheim Pharmaceuticals, Inc., 900 Ridgebury Road, Ridgefield, Connecticut 06877, United States

**Keywords:** cellular cytoplasm, protein condensation, macromolecular crowding, phase separation, molecular dynamics simulations, Brownian dynamics simulations

## Abstract

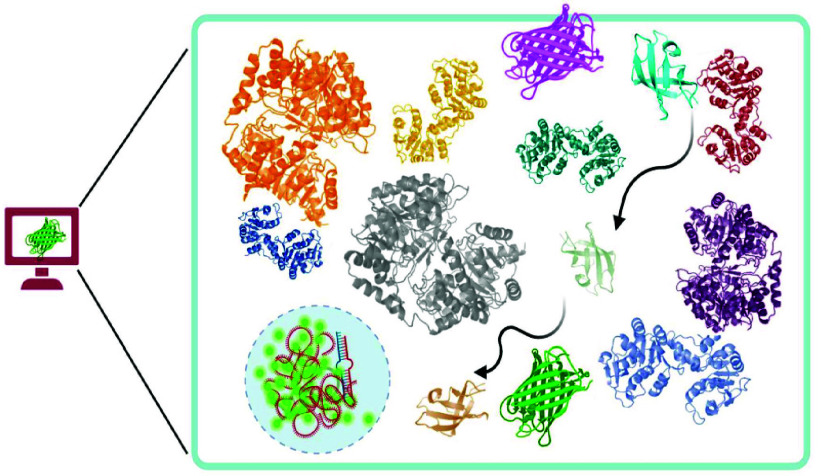

Macromolecular crowding in the cellular cytoplasm can potentially impact diffusion rates of proteins, their intrinsic structural stability, binding of proteins to their corresponding partners as well as biomolecular organization and phase separation. While such intracellular crowding can have a large impact on biomolecular structure and function, the molecular mechanisms and driving forces that determine the effect of crowding on dynamics and conformations of macromolecules are so far not well understood. At a molecular level, computational methods can provide a unique lens to investigate the effect of macromolecular crowding on biomolecular behavior, providing us with a resolution that is challenging to reach with experimental techniques alone. In this review, we focus on the various physics-based and data-driven computational methods developed in the past few years to investigate macromolecular crowding and intracellular protein condensation. We review recent progress in modeling and simulation of biomolecular systems of varying sizes, ranging from single protein molecules to the entire cellular cytoplasm. We further discuss the effects of macromolecular crowding on different phenomena, such as diffusion, protein–ligand binding, and mechanical and viscoelastic properties, such as surface tension of condensates. Finally, we discuss some of the outstanding challenges that we anticipate the community addressing in the next few years in order to investigate biological phenomena in model cellular environments by reproducing *in vivo* conditions as accurately as possible.

## Introduction

While computer simulations to investigate biomolecular behavior are usually performed in model *in vitro* conditions, biological phenomena happen inside a more complex environment, the cellular cytoplasm. The cell is densely packed with diverse macromolecules, including a wide variety of proteins and nucleic acids, which act as crowders and can reach intracellular concentrations as high as 300 g/L.^1^ Such crowders exert influence via volume exclusion and specific weak and transient interactions with other macromolecules, known as soft or quinary interactions.^2,3^ Additionally, the presence of large immobile macromolecules and membranes can lead to physical boundaries and confinement. Notably, a major consequence of the crowded environment inside living cells is the formation of intracellular mesoscale membraneless bodies, also known as *biomolecular condensates*. Emerging evidence from recent studies indicates that biological molecules, such as proteins and nucleic acids, undergo spontaneous demixing and phase separation, which allow cells to achieve high degree of spatiotemporal control over the organization of their internal content.^4^ Examples of such protein and nucleic acid rich condensates are as varied as P-bodies and stress granules in cytoplasm, and nuclear condensates such as nucleoli and DNA repair foci.^5^ Apart from achieving controlled organization, these condensates play functionally instrumental roles in a range of biological pathways, such as ribosome biognesis, gene expression, cellular stress response and modulation of cellular signaling pathways. Most importantly, the formation of dysfunctional condensates, especially in their amorphous, solid-like phases, is frequently associated with neurodegenerative disorders^6^ and various types of cancer.^7^

Recent advances in experiments and simulations are paving the way toward a future where simulating cellular environments is becoming feasible. From the experimental side, proteomics data provide detailed information about the types of macromolecules and their abundance inside prokaryotic and eukaryotic cells, while techniques such as cryo-electron tomography (cryoET) and cryo-electron microscopy (cryoEM) allow the elucidation of the structure of large macromolecular complexes. On the computational front, AlphaFold2^8,9^ and AlphaFold3^10^ have demonstrated the potential to accurately model proteins and protein complexes without the need for experimental structures. Additionally, the advent of graphics processing units (GPUs) has significantly boosted computational power, enabling the simulation of larger and more heterogeneous systems. Recent works have highlighted how data from electron microscopy and other experiments can be combined with AI-based structure prediction tools to obtain information about proteins in the cellular environment, thereby providing new insights about intracellular spatial organization and native interactions.^11,12^

Over the last three years (2021 onward), the period of focus of this review, numerous computational strategies have been developed by various research groups to model and simulate crowded cellular environments and biomolecular phase separation and condensation inside cells. These models vary significantly in scale, ranging from single-protein systems to multicomponent ones, leading up to models encompassing all the biomacromolecules within a bacterial cytoplasm. Our goal with this review is to compile those computational methods which have been developed in the past few years for simulating biomolecular crowding and condensation in cellular environments. We aim to encourage progress toward a future where these methods can be collectively implemented to simulate systems that mimic cellular environments as closely as possible. This approach could lead to a deeper understanding of how crowding and the dense cellular medium influence biomolecular events. With this aim in mind, we have described in this review the models and systems used to investigate cellular crowding and condensation, the computational methods employed to simulate these systems, and the key mechanistic insights gained from these models and simulations.

In this brief review, it is not possible to describe in detail all the computational methods and the systems to which such methods were applied. We refer the reader to a few of the reviews which have been published in the past few years with a detailed discussion on crowding, biomolecular condensates and computational methods to investigate crowded cellular environments and condensates.^4,13−20^ Here, we have dedicated two separate sections to the modeling and simulation of intracellular crowding and protein phase separation and condensation inside cells. While both topics constitute the subject of biomolecular crowding within cells, we chose to separate them to discuss the specific modeling and simulation methods developed for each type, as these fields have evolved rather independently over the years. By addressing these two areas together in this review, we aim to encourage a holistic approach to modeling of intracellular crowding.

## Cellular and Crowded Environments

### Structural Models

**Figure 1:**
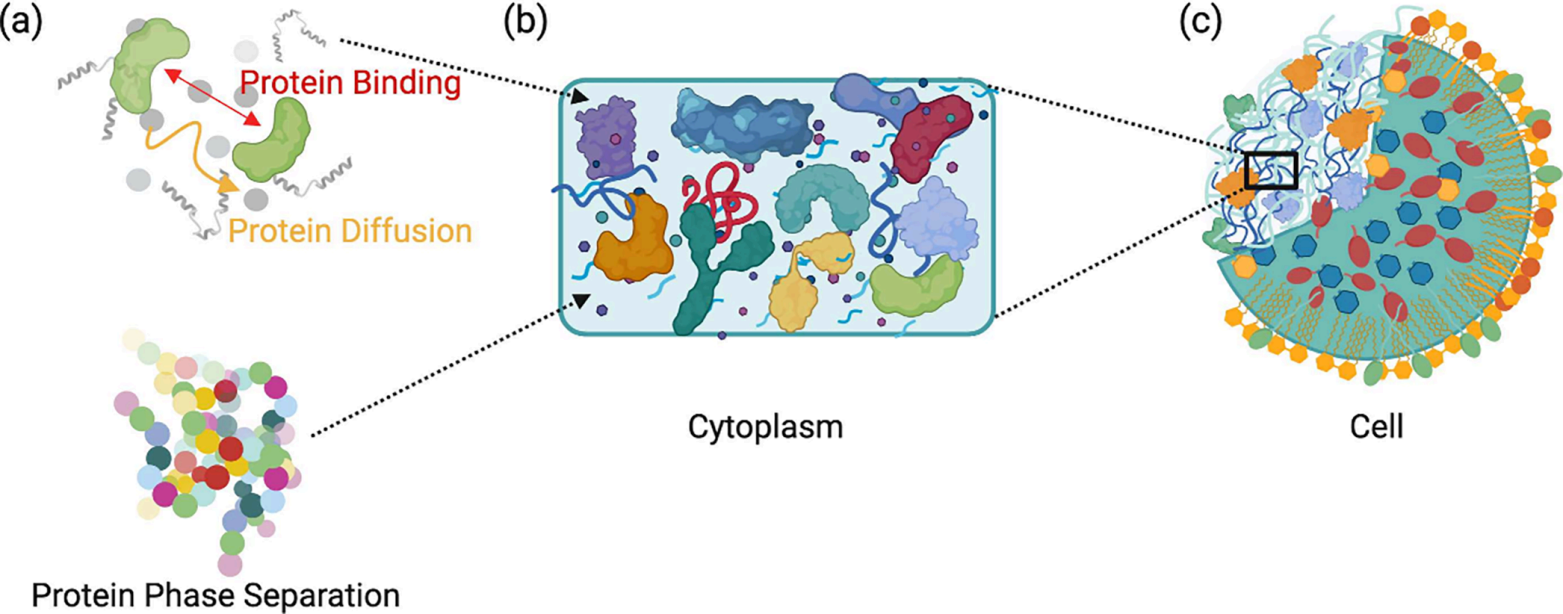
Macromolecular crowding within cells dictates various cellular functions, including protein diffusion, protein–protein interactions, protein–ligand association/dissociation, and protein phase separation or condensation. This review explores the molecular modeling and simulation methods developed in past few years to study the effects of macromolecular crowding across various spatiotemporal scales. These methods range from modeling the effect of crowding on protein multimers, understanding the molecular mechanism of protein condensate formation (a) to simulating the cytoplasms of multiple organisms (b) and developing structural models of entire cells (c).

In this section we discuss the recent works which have developed methods to model biomolecular crowding and crowded cell-like environments. The models vary from simple systems, with one type of protein crowder, up to large, heterogeneous systems containing all the elements of a prokaryotic cell, as summarized in Figure 1.

At a fundamental level, the effect of the complex and heterogeneous cellular environment can be loosely mimicked by constructing models of simple crowded systems, with high concentrations (tens to hundreds of g/L) of one type of crowding agent. In this direction, the simplest *in-silico* models have used synthetic, nonbiological crowders like polyethylene glycol (PEG), Ficoll or fullerene molecules.^21−25^ These crowders reproduce the effect of volume exclusion, as can be expected with biomolecules that contribute to intracellular crowding. However, since they are mostly chemically inert in nature, the auxiliary effects caused by weak quinary interactions between crowders and a test protein usually cannot be captured in this case. Although, a very recent work^22^ has challenged this hypothesis and proposed that there are specific chemical interactions between PEG and proteins, which should be considered to understand the molecular level effect of PEG-induced crowding.

Nevertheless, to represent both volume exclusion and weak interactions induced in a crowded environment, protein crowders are used more commonly. Proteins such as bovine serum albumin or hen egg white lysozyme^26,27^ are typically used due to the availability of experimental data, since these proteins can be easily extracted in high concentration, which is required for an experimental set up. Studying crowding using only one kind of protein crowder offers the advantage of system simplicity and the possibility to gain detailed mechanistic insights. However, since these systems contain only a single type of crowder and, therefore, a limited variety of weak interactions with the test protein, they do not constitute a true depiction of *in vivo* conditions.

Due to these reasons, attempts have been made to increase system complexity further by constructing heterogeneous crowded environments, by adding different protein crowders, in order to replicate the cytoplasm of different cell types as closely as possible.^28,29^ The *E. coli* cytoplasm has been a popular model system in this regard, due to the availability of many experimental data, like crystal structures of proteins, proteomics studies and diffusion rates.^30^ The pioneering work by McGuffee and Elcock^28^ was the first one to propose a fully atomistic model of the *E. coli* cytoplasm, where the system was composed of 50 types of protein, which led to a macromolecular concentration of 275 g/L. This model has since been adopted by many crowded environment studies. Other pioneering studies investigating macromolecular motion and diffusion in crowded cell-like environments include the studies of Ridgway et al.^31^ and Ando and Skolnick.^32^ Since this review focuses on works published in the field in the last 4 years, several noteworthy simulations of the cellular environment published before 2020 are not covered here, but have been discussed by Feig et al.^33^ and Grassmann et al.^17^ In a more recent study, Rickard et al.^29^ have developed a simplified atomistic model of the *E. coli* cytoplasm, using single copies of 12 types of proteins as crowders. They have employed this model to investigate the effects of a crowded cytoplasm on the conformations of ATP molecules, a key metabolite present in high concentrations within cells. In a similar work, Timr et al.^34^ have used coarse-grained simulations to develop a heterogeneous model of the *E. coli* cytoplasm with 35 protein families and a total of 197 proteins in order to investigate the effect of crowding on the thermal stability of proteins.

With the advent of more powerful computational resources, the scientific community has made significant progress toward constructing more realistic computational models of intracellular crowded environments. As a latest part of the progress, structural models of entire cells are being developed. At this stage, most of the models are restricted to prokaryotic organisms. In fact, the most popular organism adopted as a model system for complete cells is the *Mycoplasma genitalium*, due to the small cell size and limited number of proteins.^35−37^ One of the earliest large-scale atomistic simulations was performed by Yu et al, who simulated a model of a portion of the Mycoplasma cytoplasm.^38^ A pioneering work in this direction has been done by Maritan et al.,^37^ who have built the first structural model of the entire cell of *Mycoplasma genitalium* using a combination of macromolecular modeling and visualization softwares designed specifically for proteins, lipids and nucleic acids, such as CELLPACK, LatticeNucleiod, LipidWrapper and Mesoscope.^39,40^ The authors have first constructed a coarse-grained (CG) model of the Mycoplasma cell, followed by which the atomistic coordinates of the biomolecules have been mapped on to the corresponding CG beads to build a holistic, atomistic bacterial cell model.^37^ The first minimal synthetic bacterial cell, JCVI-syn3.0, a derivation from Mycoplasma, is also being used for 3D cell modeling studies.^41,42^ A representative CG model of the JCVI-syn3.0 cell, composed of all the subcellular compartments, has been developed by Stevens et al. using CG-based modeling tools like Bentopy,^41^ Polyply^43^ and TS2CG^44^ of the Martini modeling ecosystem.^41^ It should be noted, however, that these models of entire cells have not been simulated so far due to the challenges that the current simulation tools encounter while dealing with large system size, as discussed by Stevens et al.^41^

While most models available so far are for prokaryotic cells, some models of more complex eukaryotic cells are also available. A human cytoplasmic system made of 10 protein families was constructed by Russell et al. to study macromolecular dynamics in cells.^45−47^ The main goal of these studies is to build initial structures of cells or portions of the cytoplasm, which can be used later to study the dynamics of proteins of interest in native cellular environments as well as characterize the complex molecular interactions that drive different biochemical processes within cells. The key challenges still lie in the lack of visualization, simulation and characterization methods of such large, dense and complex models.

### Computational Methods

**Figure 2:**
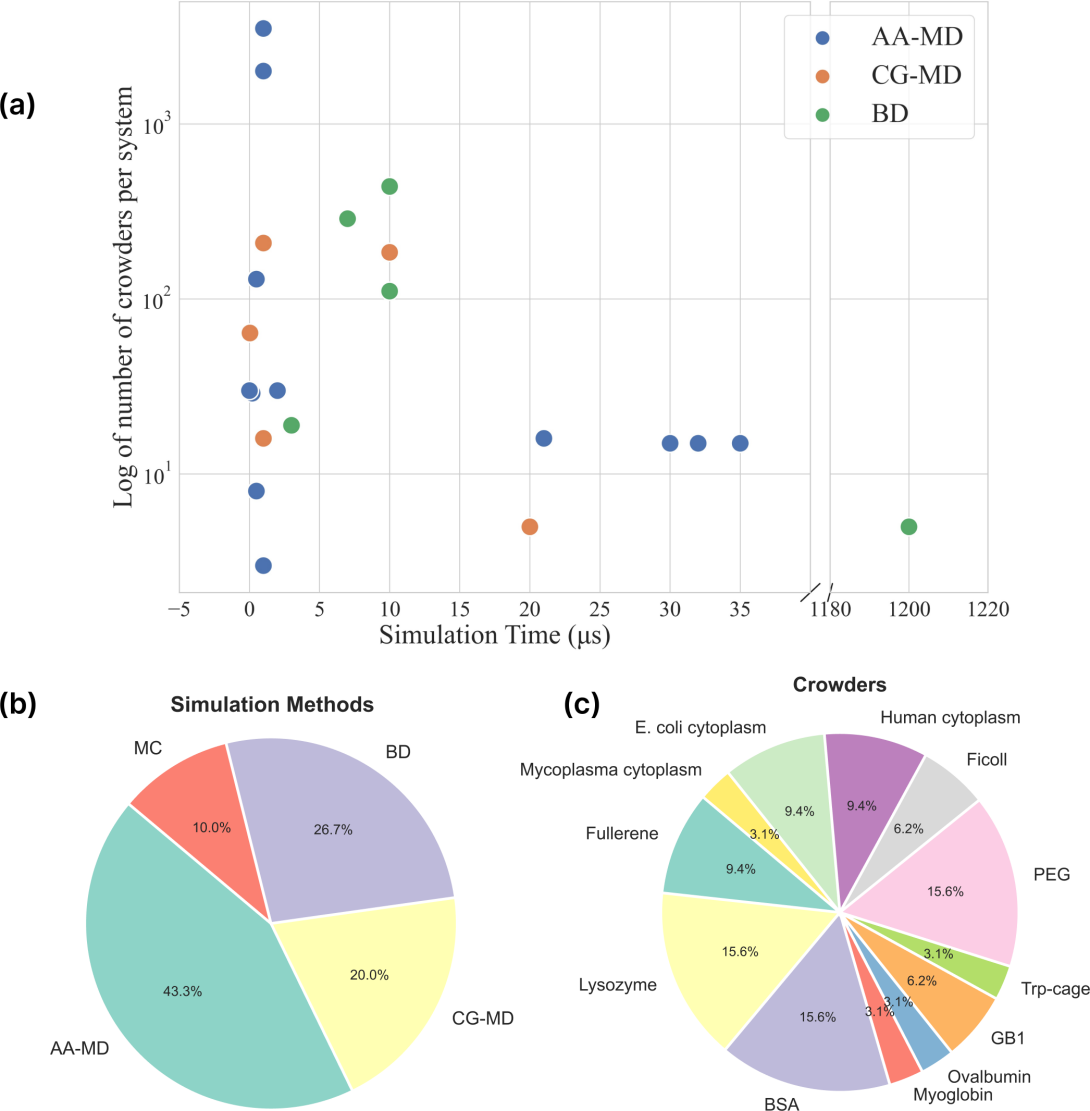
Simulation time as a function of the system size (a), statistics of methods (b) and protein crowders or systems (c) used in recent simulations employed to study crowded and cell-like environments. Data obtained from the studies reported in Table 1. (a) The graph shows the log of the number of crowders used in simulations versus the total time of simulations. Smaller systems are typically simulated for longer time scales due to computational costs. AA-MD: all-atom molecular dynamics; CG-MD: coarse-grained molecular dynamics; BD: Brownian dynamics. (b) Statistics of methods used by works discussed in the review in percentage: Monte Carlo (MC), Brownian dynamics (BD), coarse-grained molecular dynamics (CG-MD) and all-atom molecular dynamics (AA-MD) simulations. (c) Statistics of the usage of different types of crowders or systems discussed in the review in percentage. BSA: bovine serum albumin, PEG: polyethylene glycol.

The models of cellular cytoplasms and crowded environments described in the previous section, in combination with simulations, have been used to provide mechanistic insights about the effects of crowding over biological phenomena. However, due to the challenges in the simulation of large and heterogeneous environments, different studies have focused on distinct aspects of crowding effects, using different system sizes and levels of resolution. On one hand, atomistic simulations provide in-depth molecular-level insights into conformational dynamics and interactions of proteins in crowded environments. On the other hand, lower resolution simulation techniques, from coarse-grained simulations to Brownian dynamics or Monte Carlo simulations, allow the investigation of longer time scales, while compromising on the smaller details. Here, we briefly introduce and discuss the applications of different simulation techniques in recent years, which are also summarized in Table 1 and Figure 2.

**Table 1:** Summary of Methods, Simulation Time and Timestep, Number of Crowders or Intrinsically Disordered Proteins (IDPs), Types of Metabolites and Ion Concentration of the System, Software, Force Field, Water Model, and Properties Investigated in Recent Simulations Employed to Study Crowded and Cell-Like Environments and Biomolecular Condensates

Method	Total simulation time (μs)	Simulation timestep (ps)	Total number of crowders/IDPs^a^	Metabolites/ion (conc. in mM)	Software	Force field^b^	Water model^c^	Properties observed	Ref.^d^
Crowded Environment									
BD^e^	2 × 10^6^	1.5 × 10^9^	NA^f^	-	SMOLDYN	-	-	Diffusion	(58)
BD	1200	1	5	-	READDY	-	-	Dimerization	(56)
BD	10	0.5	111	-	BD_BOX	-	-	Diffusion	(68)
BD	10	0.5	440	-	SDA7	-	-	Diffusion	(26)
BD	7	0.1	76, 76, 152, 228, 76, 73, 66	-	pyBrown	-	-	Diffusion	(66)
BD	5	0.5	NA	-	SDA7	-	-	Diffusion	(65)
BD	3	0.5	19	-	Geom3d	-	-	Diffusion	(60)
BD	NA	NA	NA	-	MATLAB Custom script	-	-	Diffusion	(70)
CG-MD^g^	20	0.4	5	NaCl (NA)	GROMACS	MARTINI	-	Dimerization	(56)
CG-MD	10	0.2	31, 60, 90, 122, 185	-/NaCl (150)	GROMACS	MARTINI	-	Ligand binding	(90)
CG-MD	1, 1	0.1, 0.2	9, 64, 45, 209, 209	-	MUPHY	OPEPv7	-	Protein stability	(34)
CG-MD, AA-MD	1, 1	0.1–0.2, 0.02	CG-9, 64, 45, 209, 209; MD-1, 3	-/K (NA)	MUPHY, GROMACS	OPEPv4, AMBER99SB-ILDN	-, TIP3P	Protein stability	(77)
CG-MD	0.04	1	64	-	GROMACS	SMOG	-	Protein stability	(72)
CG-MD	NA	NA	NA	-	in house software	-	-	Protein stability	(78)
AA-MD^h^	35	0.02	10, 15	Amino acids (GLU, ASP, GLN, GLY, GSH, ASX, ALA, SER, Ac-SER, LEU, ILE, THR, ARG, HIS, VAL, TYR, B-ALA, HYP), ATP, UTP, GTP, CTP, ADP, GDP, UDP, UDP-hexose, AMP, GMP, CMP, NAD, GSSG, G3P, G6P, 3PG, dTTP, GABA, Malate, Spermine, Spermidine, Lactate, Taurine, (Iso-)Citrate, Putrescine, Phosphocholine, 3-Hydroxybutyric acid, Fumarate, FBP, Succinate, Pyruvate, Ribose-5-phosphate, Pantothenic Acid, Citrulline, Phosphocreatine, Creatine, Alpha- ketoglutarate, Choline, Adenine, Carnitine, Glycerophosphocholine/K (157.3, 155.6), Na (4.25, 3.87), Mg (8.50, 8.39), Cl (11.0, 5.16)	NAMD	CHARMM36	TIP3P	Protein stability, domain motion	(47)
AA-MD	32	0.02	10, 15	-	NAMD	CHARMM36	TIP3P	Complex formation	(45)
AA-MD	30	0.02	10, 15	-	NAMD	CHARMM36	TIP3P	Protein stability, folding	(46)
AA-MD	21	0.01	16	Amino acids (GLU, ASP, GLN, GSH, ALA, MET, LEU, LYS, ARG, VAL, HCY), ATP, UTP, GTP, CTP, GDP, UDP, UDP-glucose, UDP- GlcNAc, UDP- glucuronate, AMP, IMP, NAD, GSSG, G6P, 3PG, 6PG, dTTP, FAD, DHAP, FBP, Acetyl-CoA, CoA, Gluconolactone, Acetylphosphate, Pentose-*Pc*, Citrulline, Glycerate, Malate, Citrate, Uridine, Hexose-Pa, Succinate/K (300, 300), Na (25.5, 40.4), Mg (34, 36.5), Cl (13.6, 5)	NAMD	CHARMM36-mCUFIX	TIP3P	Small molecule stability	(29)
AA-MD	2, 1	0.02	5, 10, 30, 3, 9, 13, 2, 2, 2, 4, 2, 6	sucrose, urea/-	OPENMM	CHARMM36m	TIP3P	Diffusion, protein stability and interactions	(91)
AA-MD	1	0.02	2, 4, 2008	-/NaCl (150)	GENESIS	CHARMM36	TIP3P	Diffusion, ligand binding	(27)
AA-MD	1	0.01	270-3517	-/NaCl (100)	Desmond	AMBER99SB-disp	a99SB-disp	Protein stability, aggregation	(25)
AA-MD	0.5	0.01	1, 8	Glycerol/NaCl (NA)	GROMACS	CHARMM22	TIP3P	Protein stability	(82)
AA-MD	0.5, 1	0.02	130, 16	-/NaCl (20)	NAMD, OPENMM	CHARMM36	TIP3P	Diffusion, protein stability	(23)
AA-MD	0.5	0.02	130, 110	-/NaCl (20)	NAMD, OPENMM	CHARMM36	TIP3P	Diffusion, protein stability	(24)
AA-MD	0.25	0.01	11	Glycerol, spermidine, osmoregulated periplasmic glucan/KCl (150), Ca (NA)	GROMACS	CHARMM36m	TIP3P	Ligand binding	(83)
AA-MD	0.20	NA	1, 8, 8, 7, 29	Amino acids (ALA, ASP, GLU, GLN, GLY, SER, THR, UD1), ATP, Pyruvate/Na (NA), K (NA)	AMBER18	AMBER14SB	NA	Ligand binding, ligand stability	(92)
AA-MD	0.005	0.02	15, 30, 2	Sucrose/MgCl (2)	NAMD	CHARMM27	TIP3P	Diffusion, polymerization	(21)
Biomolecular Condensates									
CG-MD	27–40	20	1–2	-/NaCl (NA)	GROMACS	MARTINI3	-	SAXS, PRE, dimerization	(93)
CG-MD	2.5–20	20	NA	-	OPENMM	COCOMO (LD)^j^	-	Clustering, phase separation	(94)
CG-MD	12	30	50–672	-	GROMACS	MARTINI2	-	Surface tension, shear viscosity, phase diagram	(95)
CG-MD	10	20	100	-	LAMMPS	LJ (LD)	-	Self-assembly, orientational order parameter, disorder to order transition	(96)
CG-MD	5	10	100	-	LAMMPS	HPS^i^ (LD)	-	Diffusion, second virial coefficient	(97)
CG-MD	0.155, 0.2, 2, 3.5	10	2, 100, 300	-	HOOMD-Blue, OpenMM	HPS (LD)	-	Second virial coefficient, phase diagram	(98)
CG-MD	2.5	20	50	-/NaCl (50)	GROMACS	MARTINI3	-	Surface tension, aggregation behavior	(99)
CG-MD	0.5–2.5	10	NA	-	HOOMD- Blue, LAMMPS, GROMACS	HPS (LD), MARTINI	-	Diffusion, viscosity, surface tension	(100)
CG-MD	NA	NA	NA	-	HOOMD-Blue, LAMMPS	HPS-Urry framework (LD)	-	Phase behavior	(101)
CG-MD, AA-MD	10, -	15, -	50–100	-	LAMMPS, NAMD	LJ (LD), -	TIP3P	Self-assembly, diffusion	(102)
AA-MD, CG-MD	≈ 12, 5	2, 10	NA, 100	-/NaCl (100)	HOOMD- Blue, LAMMPS, GROMACS	HPS (LD), -	TIP4P/2005	Radius of gyration, coexistence densities, pairwise contacts	(103)
CG-MD, AA-MD	1, 0.1	20, 2	8	-/NaCl (150)	GROMACS	AMBER99SB-Disp, MARTINI3	a99SB-disp	Interaction energy, water entropy, protein conformation, entropy	(104)
AA-MD, CG-MD	0.3, 2	-, 15	4, 48	-/NaCl (NA)	GROMACS, LAMMPS	AMBER99SB-Disp, Mpipi	TIP3P	PMF, coexistence density, aging	(105)
AA-MD	0.12–0.4	2	NA	-/NaCl (150)	GROMACS	AMBER99SB-ILDN	TIP4PD	Conformational properties, dimerization, effect of phosphorylation	(106)
AA-MD	0.2–0.5	2	125, 125, 343	-/NaCl (150)	GROMACS	CHAARMM36m	TIP4P/2005	Spin relaxation rate	(107)

a Number of crowders are reported for crowded environment simulations and number of intrinsically disordered proteins (IDPs) are reported for biomolecular condensate simulations.

b Force field is only mentioned for coarse-grained and all-atom molecular dynamics simulations.

c Water model is only mentioned for all-atom molecular dynamics simulations.

d Reference.

e Brownian Dynamics.

f Information not available.

g Coarse-grained molecular dynamics.

h All-atom molecular dynamics.

i Hydropathy scale.

j Langevin dynamics.

#### Monte Carlo Simulations

In Monte Carlo (MC) simulations, starting from an initial configuration of the system, a move that changes the configuration is attempted. Such a move is accepted or rejected based on the Metropolis criterion. This guarantees that the configurations are sampled with the correct statistical weight in the simulation, allowing one to investigate protein conformational changes and to compute average thermodynamic properties in crowded environments. In MC simulations, the crowders can be either considered inert, or defined to have specific interactions with the target protein.^48−50^ Inert crowders have only a steric effect, which was shown by Wang et al.^49^ to be a major contributor in the polymerization process. The authors compared MC simulations of the bacterial tubulin proteins BtubA and B in the presence of sticky and nonsticky crowders, and postulated that exogenous proteins could also function in cells even though the local tertiary interactions changed due to the volume exclusion effects of crowders. In a different study, the presence of protein-crowder interactions led to the appearance of a new, crowder-assisted pathway for the formation of the complex between the protein and its target DNA sequence.^50^ In another example, using a lattice-based kinetic MC simulation, which uses the transition rates among states in the system as input to enable simulation of dynamic behavior, a simulation of 2000 proteins with 40000 crowders for 200 s was performed by Basu et al.^48^ However, one of the drawbacks of this method is that no dynamical information can be gathered from conventional MC simulations.^51^ This is in contrast to MD simulations, where dynamical information can be obtained deterministically due to the use of Newton’s equation to propagate motion.

#### Brownian Dynamics Simulations

A popular mesoscopic method to study crowded environments is Brownian Dynamics (BD) simulations, where the motion of particles is described by overdamped Langevin dynamics, and a stochastic force is used to represent collisions with water, which is represented implicitly. This method is very versatile in terms of biomolecule representation, and number and type of crowders.^52^ Several softwares have been developed to help prepare systems, simulate and analyze BD simulations, as summarized in a previous review.^53^ BD simulations allow for large time steps (around picoseconds) to be used to propagate motion, at the expenses of using implicit solvation and removing the internal motion of molecules. It is thus useful to achieve large simulation time scales (up to several microseconds). These simulations are helpful to record bulk properties such as diffusion, protein association and aggregation, which are hard to observe in classical MD simulation time scales, since the time step of the latter is usually shorter, 1–2 fs.

Using the software ReaDDy, a particle-based reaction-diffusion simulator,^54,55^ and a system with 5 copies of a single crowder protein and 2 copies of the target protein GB1, all represented using single beads, BD simulations revealed that, while inert crowders could promote dimer association, attractive crowders with affinity for GB1 could disrupt the process.^56^ Other examples of application of BD simulations include the use of the software Smoldyn, a program for cell-scale biochemical simulations,^57^ to investigate protein diffusion rates in the *E. coli* cytoplasm,^58^ using a system with up to 500 spherical particles to represent cytoplasmic proteins. Geom3D^59^ was used to investigate substrate association in a system with 2 enzymes, their substrate and 19 inert crowders. These simulations showed that crowding reduced the diffusion rates, thereby increasing the enzyme–substrate association rates.^60^ In another example, Simulation of Diffusional Association (SDA)^61−63^ was used to simulate large systems, such as a system with 80 small molecules and 440 protein crowders, which was used to study the diffusion rates of 4 different small molecules in the same crowded environments. This study observed that, contrary to expectations, the diffusion rates of small molecules could increase in a crowded environment due to reduced aggregation or surface desorption.^26,64^ SDA was also used to investigate subdiffusion of proteins due to crowding.^65^ In an effort to study the behavior of metabolites in a crowded environment, Raczyłło et al.^66^ simulated a system with Ficoll70 as crowder and 50 metabolites represented as hard spheres of varying radii using an in house software, the pyBrown package. The simulations revealed that the metabolite diffusivity depended linearly on the fraction of volume occupied in the system. The Bd_Box software^67^ was used by Słyk et al. to investigate the effects of softness and hardness of flexible protein crowders on the diffusion of particles by modifying the attractive potentials of the crowders.^68^ Recently, Skóra et al.^136^ used a fluctuating-dumbbell model of enzymes to incorporate conformational changes in BD simulations in a simplified way. Here, the enzyme is represented by two beads, and it can be in the closed or open state, depending on the distance between the beads.^69^ Additionally, new techniques such as “Doppelganger simulations”^70^ have been developed to replicate the experimental diffusion of nanoparticles in crowded environments. By using another technique, reversible association and dissociation events between particles were simulated to show the improved rebinding of ligands to target proteins due to the cage effect of crowding, which is characterized by transient confinement of a particle by its neighboring particles.^71^

#### Coarse-Grained MD Simulations

To gain further molecular insights without demanding high computational resources, coarse grained (CG) representations of the macromolecules can be used in combination with molecular dynamics (MD) simulations. Here, the crowder proteins and test proteins can be represented by one or more beads, and water may be represented as beads or implicitly. Simulations with a CG force field can achieve long time scales as compared to atomistic simulations. At a lower resolution, where each protein is a spherical particle, a longer time step of 1 ps can be used, allowing one to simulate systems up to milliseconds.^56^ At higher resolution, where every amino acid in a protein is represented as one bead, the time step is usually shorter, around 1 fs.^34,72^ In an effort to improve the performance of large-scale CG MD simulations, a unique domain decomposition scheme with dynamic load balancing was implemented by Jung et al. in the Generalized-Ensemble Simulation System (GENESIS) software package^73−75^ as an MD engine called CGDYN.^76^

The effects of the crowded environment on the stability and folding of proteins,^72,77^ dimer formation^56^ and fold switching^78^ have been studied using CG force fields like Martini,^79^ OPEP^34^ and SMOG.^80^ Bazmi et al. used potential functions to describe and alter protein-crowder interactions. Langevin Dynamics combined with coarse-grained models of protein G and crowders revealed how volume exclusion provides stability to all folded states.^78^ Additionally, CG MD simulations were combined with enhanced sampling methods to further explore the effects of crowded environments on protein–ligand binding and protein folding. For example, to improve sampling of protein dimerization, Pradhan et al. used well-tempered metadynamics simulation combined with the parallel tempering (PTMetaDWTE) method.^56^ Destabilization of the protein dimers in the presence of lysozyme as protein crowder was observed.

One of the advantages of CG MD simulations over typical rigid body BD simulations is that protein conformational changes can be sampled more extensively in CG MD simulations. However, while the dynamics of proteins can be observed at lower computational demand with CG force fields, detailed molecular interactions, such as hydrogen bonds, are still missing.

#### All-Atom MD Simulations

All-atom (AA) MD simulations have been used to investigate a variety of crowded systems over a large range of simulation times. The time scales and system sizes associated to MD simulations have increased manifold in recent times due to the speed up in calculations provided by GPUs, usage of high performance specialized supercomputers, such as Anton2,^81^ and specialized software, such as the GENESIS software package.^73−75^ Kasahara et al. simulated the binding of the Src kinase and its inhibitor in diluted and crowded conditions, using bovine serum albumin as a protein crowder, for 100 μs with Anton2 supercomputers.^27^ AA MD simulations were also employed to study the stability of macromolecules and polymerization processes in the presence of crowders and osmolytes.^25,77,82,83^ Timr et al. investigated protein thermal stabilization in crowded environments using the OPEP force field and a hybrid particle–lattice approach called Lattice Boltzmann molecular dynamics (LBMD) simulation.^34^ Using a multiscale approach, snapshots of CG MD simulations were used to perform AA MD simulations using the Replica Exchange with Solute Scaling 2 (REST2)^84,85^ enhanced sampling method to investigate protein unfolding events.^77^

The choice of water model can impact diffusion rates and protein conformations in simulations. Abriata et al.^86^ and Sarthak et al.^87^ investigated the effects of different water models in the aggregation of intrinsically disordered proteins. While the simulations by Abriata et al. suggest that force fields combined with three-point water models, CHARMM36m-TIP3P* and ff19SB-OPC, are able to capture the preferred secondary structure and population percentages of disordered proteins, Sarthak et al. observed that four-point water models, TIP4P-D model and its variant, TIP4P-D-1.6 (aadisp), slightly expanded the ensemble of conformations. The effects of water models in simulations of crowded systems were also reviewed by Gopan et al.^88^

Due to the overwhelming amount of data that is generated in such large-scale AA MD simulations, data analysis becomes a challenge. A set of analysis tools called Spatial decomposition analysis (SPANA) was recently developed in the GENESIS software package, with the aim of assisting trajectory analyses of such large-scale simulations.^89^

The advantages of using AA MD simulations are the higher level of detail in the representation of molecular interactions in the systems, conformational changes of molecules being fully taken into account, and binding events and their intermediate states can be studied in detail. However, MD simulations of crowded systems with AA representation have a very high computational cost, and therefore the computational resources required to run such systems are enormous.

## Phase Separation and Biomolecular Condensates

A direct consequence of intracellular crowding is the phase separation or condensation of biomolecules, resulting in the formation of membraneless organelles with regulated architecture. In fact in recent years, the field of intracellular biomolecular condensation has largely proliferated, with significant collective efforts being dedicated to understand the molecular origins of the regulated organization of materials within cells.^4,5^ This interest has intensified with the growing recognition of the crucial role these condensates play in human health and disease. Beyond their biological roles, a strong interest has developed in the community to explore how these condensates function within the crowded cellular milieu, and to identify the specific molecular interactions that underlie their dynamic and size-regulated behavior.

A significant challenge in this area is to determine a comprehensive, multiscale view of the spatial organization and dynamics of protein condensates that would connect to the conformational properties and interaction patterns of individual polypeptides within a crowded environment. Given the extreme structural and dynamic heterogeneity of these condensates, it is essential to capture the statistical, ensemble-level aspects of how molecules inside the condensates are spatially organized. Due to the inherent physical nature of the biomolecular condensation process, intrinsically complex molecular composition and stoichiometry of these condensates, molecular modeling approaches have proven to be extremely beneficial in understanding the underlying physical behavior of the condensate assembly and phase separation phenomena. Additionally, recent development of the data driven and machine learning based approaches have been quite successful in predicting the protein structural and material properties and their role in determining protein condensation behavior.

Recent progress in molecular modeling and simulation techniques has significantly deepened our understanding of the structural properties and dynamics of biomolecules, especially for those of intrinsically disordered proteins (IDPs) that predominantly constitute biomolecular condensates. These insights have proven to be particularly crucial for understanding the physical mechanisms underlying protein phase separation and determining their material and viscoelastic properties. Depending on the scale of resolution, molecular modeling excels in identifying the residue-specific interaction dynamics within proteins. This allows for the analysis of how specific intermolecular interactions and molecular composition influence the structural and dynamical behavior as well as material properties of the protein condensates. With biomolecular condensates becoming a topic of major interest, molecular simulation techniques at different resolutions are being developed and corresponding potential energy functions are being modified, in order to gain access to higher spatiotemporal scales and study molecular properties and phase separation of IDPs with increasing statistical accuracy. In this section, we discuss the recent progress made in the past few years in developing computational models and methods to investigate the molecular origins of biomolecular phase separation and to characterize their intrinsic structural and dynamic behaviors (details of the data obtained from the simulations of different molecular models are tabulated in Table 1).

### Physics Based Methods at Different Levels of Resolution

#### Coarse-Grained Modeling

Biomolecular condensate assembly and phase separation occurs at a mesoscale level, which is the scale that straddles the molecular (nanometer) and cellular (micrometer) scales. To address the challenges posed by large system sizes and long time scales at this scale, implementing coarse-grained (CG) molecular modeling methods emerges as the most effective strategy. CG modeling is essentially a zoomed out view of atomic resolution of a molecule, where multiple atoms together are represented as a single CG bead. Over the years, several strategies have been adopted by different groups in order to design efficient coarse-grained models of IDPs, thereby reproducing their structural behavior and determining corresponding characteristic mesoscale dynamic and material properties.

One of the most widely used coarse-grained force fields is the Martini, which represents an average of four atoms into one single interaction site. In older version of Martini 2,^108^ each interaction site falls into one of the four bead categories based on the chemical properties of its constituent atoms, while in the newer and improved version, Martini 3,^79^ there are seven bead categories due to the addition of three new bead types, divalent ions, halogen atoms and water, for improved chemical representation of molecules. In spite of Martini force field’s success in reproducing lipid bilayers’ properties, biomolecular simulations using Martini force field encountered several limitations over the years due to the incorrect representation of intermolecular interactions. In order to accurately model phase separation of FUS low complexity domain (LCD) using Martini 2.2, Benayad et al.^95^ effectively rescaled the protein–protein Lennard-Jones (LJ) interactions. With this readjustment of protein–protein interactions against solvation and entropic contributions, they could reproduce the experimental excess transfer free energy between the dense and dilute phases of protein condensates. In a related study, Larsen and colleagues^93^ found that the Martini 3 force field tends to underestimate the radius of gyration–a measure of a protein’s size–for several IDP sequences, including FUS LCD, when compared to the corresponding small-angle X-ray scattering (SAXS) data. Rescaling the protein–water LJ parameters by 10% greatly improved the agreement with SAXS data. They tested the effect of rescaling further by performing homodimerization simulation of multiple IDP sequences, and compared the results with paramagnetic relaxation enhancement (PRE) experiments. The rescaling of the protein–water interaction also greatly improved the agreement with PRE data. In a very recent study, Wasim et al.^99^ demonstrated that the tuning of the protein–water LJ parameters (both σ and ϵ) resulted in successful modeling of the phase separation of α-synuclein (αS), aggregation of which is linked to Parkinson’s disease. To explore how crowded cellular environment influences the phase separation of IDPs, they incorporated fullerene-based crowders in order to replicate cellular crowding *in vitro*. They found that the addition of crowders led to an upregulation of αS aggregation, which they primarily attributed to an excluded volume effect.

To accurately characterize the phase separation of IDPs, the modeling needs to incorporate multiple IDP chains and faithfully replicate their intermolecular interactions, which can be computationally demanding. One effective approach toward modeling the assembly and phase separation of IDPs involves representing each amino acid as a single CG bead within the IDP chain. Mittal and colleagues have been at the forefront of developing this class of CG models, where they define the nonbonded interactions between the CG amino acid beads based on hydropathy scales.^109^ In a follow-up study, Mittal and colleagues refined their initial model by adopting the Urry Hydropathy Scale^101^ instead of the previously used Kapcha-Rossky (KR) hydropathy scale, which could not accurately predict the experimentally observed phase separation propensities of IDPs upon mutations. In order to validate the newly optimized model, they compared the coexistence densities of two IDPs, FUS LCD and DDX4’s N-terminal domain, with experimentally observed values and found very good agreements. They also investigated the model’s ability to capture the effect of certain bulk mutations of the IDP sequences, and the changes in phase boundary upon mutation were in good agreement with experimental observations. They further implemented this model to investigate the relationship between sequence patterns in IDPs and their material properties, e.g., viscosity and surface tension, which were found to be strongly correlated.^100^ The wide variety of applicability of this model is tested by different studies,^98,103^ recent examples being the investigation of the coupling between thermodynamic and dynamic properties of the condensates as a function of IDPs’ amino acid sequence,^97^ and post-translational phosphorylation in quality controlling IDP droplets against amyloidogenicity.^102^ A different version of a similar model has been constructed in order to achieve chemical accuracy for the intermolecular behavior of IDPs, particularly for hydrophobic and electrostatic interactions such as π–π and cation-π^110^ which are known to be key drivers in the liquid–liquid phase separation of IDPs. In a new variant of residue-based CG model COCOMO (Concentration-dependent Condensation Model), developed by Feig and co-workers,^94^ both amino acid and an RNA nucleotide are represented as a single spherical bead. The corresponding nonbonded interaction parameters are adjusted according to the chemical feature of the respective CG beads. This model reproduces concentration dependent phase separation behavior of both disordered peptides and mixtures of peptides and RNA, which are well in agreement with experimental scatter plot measurements. In spite of their generic architecture, these models have been proven to be quite successful in predicting condensation of more complex systems, such as nucleoporin proteins (FG-Nucleoporins or FG-Nups), which are lined along the central channel of nuclear pore complexes (NPC),^111^ and liquid–liquid phase separation (LLPS) of chromatin driven by the intrinsic nucleosome plasticity.^112^ The model of FG-Nups^111^ is designed to be an implicit solvent model of IDPs, acronymed as 1BPA (one-bead-per-amino-acid), where the force field includes backbone stiffness of the disordered proteins from experimentally derived Ramachandran data, and the nonbonded interactions are represented as a combination of hydrophobic, electrostatic and cation−π terms. In order to explore LLPS propensity of chromatin, the oligonucleosome systems are coarse-grained at a resolution of one bead per amino acid for the protein, and the DNA at a resolution of one bead per base pair. The chemically specific detail included in this model is found to retain the physicochemical properties of the different nucleosome types within chromatin and can take into account the effect of amino acid point mutation on chromatin organization behavior.^112^

To explore the physical principles underlying biomolecular condensation process, another popular approach has been to perform molecular simulations at a phenomenological level. This class of models take a more minimalist approach by implementing beads-on-a-string designs that lack chemically specific information about the amino acid residues and are rather composed of coarse-grained beads with distinct interaction parameters. Dissipative particle dynamics have been used in multiple studies to understand the fluid network in the dense phase of model IDPs,^113^ effect of macromolecular crowding on condensate phase behavior and internal structure,^114^ and membrane curvature sensing behavior and endocytosis induced by model condensates.^115,116^ In these studies, the IDPs are represented as linear polymers composed of different hydrophilic beads, each displaying distinct interaction patterns. In a separate study, the dynamics of IDP assembly and the transition from disordered to ordered states in FUS were investigated by coarse-graining its intrinsically disordered architecture into a cluster of effective interaction sites.^96^ In this model, the coarse-grained resolution for the two distinct domains of FUS – prion like domain (PLD) and the RNA binding domain (RBD) – are chosen to be different. While the 160 residue long PLD of FUS is modeled through 20 effective interacting beads, the arginine-, glycine-rich region (RGG) of the RBD is represented at a resolution of 5 amino acids per bead. A similar model is used to monitor the thermodynamic conditions governing the transition of homogeneous FUS condensates to multiphase, heterogeneous assembly. In this case, a 20-bead long Lennard–Jones (LJ) polymer is designed to represent the FUS architecture, where one bead is equivalent to approximately 26 amino acid residues.^105^ This approach is particularly useful for understanding complex system behavior, such as membrane remodeling by biomolecular condensates. Primarily designed to replicate *in vitro* biophysical experiments, these models have been effectively implemented to study the onset of endocytosis and exocytosis of model coacervates.^117^

#### All-Atom Simulations

While advanced experimental techniques such as NMR spectroscopy, single-molecule FRET, SAXS, small-angle neutron scattering (SANS) as well as CG molecular modeling are routinely employed to explore the collective structural and dynamical behavior of IDPs, accurately measuring their molecular motions at an atomic resolution and determining the conformational heterogeneity associated with their structural disorder continue to pose significant challenges. All-atom simulations hold great potential in addressing this issue by providing the most detailed representation of any molecular systems. However, their implementation for estimating collective behavior of phase separating IDPs is rather restricted as the associated large system size and long time scales make these simulations computationally extremely demanding. Therefore, the use of atomistic simulations for these systems has been largely limited to generating starting conformations for CG simulations^118^ or mapping interaction behavior for designing CG models^110^ for a long period of time.

Over the past two to three years, there has been an intriguing shift in this approach, as several research groups started implementing all-atom simulations in order to develop understanding of various thermodynamic and kinetic aspects of LLPS in IDPs, such as, their solvation behavior,^104^ effect of post translational modification on condensate assembly,^106^ intrinsic dynamics of IDPs within phase separated condensates,^119^ to name a few. In the first instance, Mukherjee and colleagues estimated how the entropy gain from the water released during FUS-LCD assembly can thermodynamically drive their phase separation pathway.^104^ In order to understand the effect of phosphorylation on FUS-LCD oligomerization, Lao and co-workers performed replica exchange with solute tampering (REST) simulations and demonstrated that phosphorylation impede FUS dimerization, and potentially disrupts FUS fibrillar structure.^106^ While CG models with reduced dimensionality are effective for estimating mesoscale properties of condensates (e.g., surface tension and viscosity), it is rather challenging to use the coarse representations of IDPs in order to gain molecular level insight into the dynamic behavior of IDPs within their phase-separated condensates. In order to address this issue, Schuler and colleagues^119^ conducted massive, large-scale all-atom simulations to study the conformational features of two intrinsically disordered human proteins—histone H1 and its nuclear chaperone, prothymosin-α (ProTα), which are oppositely charged and undergo phase separation into a protein-depleted dilute phase and a protein-rich dense phase at high concentration. The analyses from the MD simulations are supported by single-molecule Förster resonance energy transfer (FRET) measurements, revealing that despite the high bulk viscosity of the dense phase, proteins within this phase exhibit highly dynamic behavior which is characterized by transient multivalent interactions between the oppositely charged constituents. In a similar study, the dynamic behavior of intrinsically disordered domain of the nucleoprotein of measles virus *N*_TAIL_ was explored at an atomic resolution in its dilute and condensed solution phases, respectively.^107^ The NMR relaxation rates of the protein were estimated from the corresponding MD trajectories in both the phases at three different concentrations and were validated against corresponding chemical shifts from NMR relaxation data. While it was observed that the local conformational sampling of the backbone was more or less preserved regardless of the solution density, the backbone dihedral angle dynamics and collective, chain-like motions of the IDPs were drastically slowed down in the dense phase. All-atom MD simulations aided with nonboltzmann, biased techniques also have the potential to determine the full conformational free energy landscape associated with disordered structure of IDPs. To gain insight into the complex conformational ensemble of unstructured proteins, Li et al.^120^ utilized multiple enhanced sampling techniques, including bias-exchange metadynamics and parallel-tempering well-tempered metadynamics in order to analyze the structural behavior of an archetypal intrinsically disordered protein (IDP), DHH1. Jung et al. used the GENESIS engine to simulate the fusion of IDP droplets, study the associated shape changes and ’mixing’ of constituents.^76^

### Data Driven Approaches to Predict Protein Phase Separation

With the emerging application of machine learning and artificial intelligence in life sciences over the past few years, there has been significant progress in understanding the phase behavior of protein condensates through data-driven modeling approaches. One major drive in this regard has been to develop machine learning based predictor tools in order to predict phase separation propensity of proteins by utilizing the ideas from their known features. The importance of amino acid sequence of IDPs in governing their condensate forming tendency has been widely postulated based on their electrostatic interaction behavior (π–π, cation-π, hydrophobic interactions) or patterning of their low complexity region. To find a general rule in this regard, Saar and co-workers developed the algorithm DeePhase^121^ for predicting LLPS propensity from the amino acid sequence of IDPs. To develop the algorithm, they initially gathered data from the publicly accessible LLPSDB database^122^ and identified a pattern among proteins with high LLPS propensity, characterized by amino acid sequences that are hydrophobic and predominantly disordered, with lower Shannon entropy, and enriched with polar residues. They used the derived knowledge about the sequence-specific features of IDPs as well as implicit protein sequence embeddings generated by a language model to construct machine-learning classifiers, which they used to identify LLPS prone protein sequences from the human proteome at a high accuracy. A similar LLPS prediction tool, PSPredictor, based on the amino acid sequence of proteins, was developed by Chu et al.^123^ which is reliant on features derived from protein specific language models, specifically the word2vec model. The data-driven approach has been applied to various other predictive analyses, including the design of predictive hydrophobicity scale, which can predict the phase separation properties of a protein based on its amino acid sequence.^124^ In an attempt to find the thermodynamic–dynamic trade off behavior in protein condensates, Jacobs and co-workers^97^ implemented a combination of Bayesian optimization techniques with supervised machine learning models to design new polypeptide sequences and predict the physical properties (e.g., second virial coefficient *B*_2_ and self-diffusion coefficient) from their amino acid sequence features. Afterward, they validated these predictions by determining the phase separation propensity of these polypeptides through large scale MD simulations using a CG model that has been discussed previously.^101^ A recent breakthrough in this direction has come through the work of Larsen and colleagues,^125^ in which they have tested the predictive power of an optimized, transferable CG molecular model CALVADOS in order to generate conformational ensembles for all the IDP sequences in the human proteome. They established structure–function relationship for all the IDPs analyzed from their conformational ensembles and used this knowledge to train a machine-learning model to predict protein compaction propensity from the respective amino acid sequences. A general outcome of this work is the conserved conformational properties of the orthologues of human proteins.

## Mechanistic Insights Obtained from Simulations

**Figure 3:**
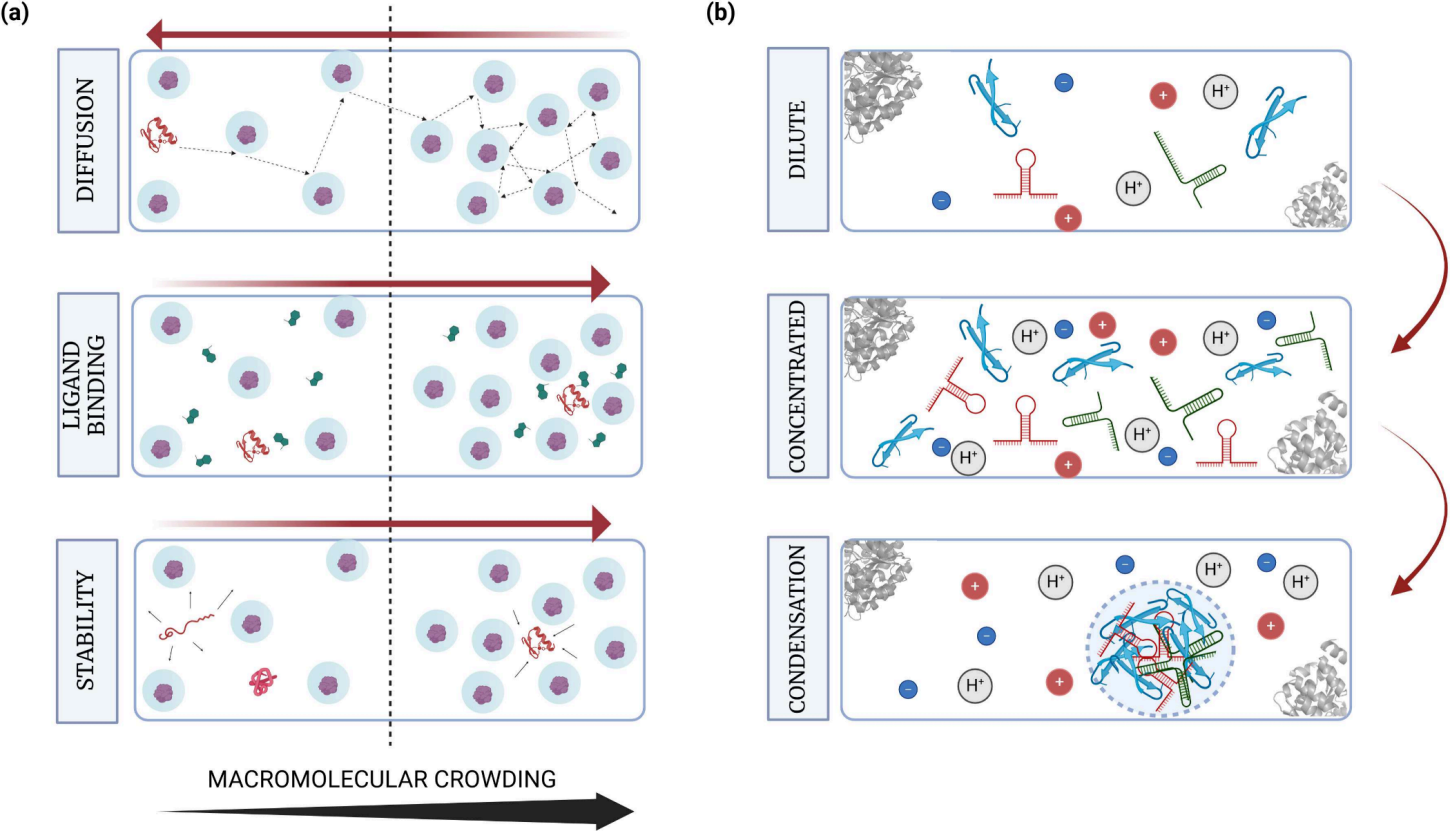
Properties investigated in simulations of crowded environments (a), cytoplasms and biomolecular condensates (b). (a) The excluded volume and quinary interactions reduce the diffusion rates of proteins in the cytoplasm. Binding of ligands to their target proteins is facilitated as the effective concentration of the ligand increases in crowded conditions. Similarly, protein structures are more stable and compact as the volume available for unfolding of proteins reduces in crowded conditions. (b) Phase separation of cellular materials to form biomolecular condensates can be facilitated by multiple physical parameters, ranging from the nature of the amino-acid sequence of biomolecules to the pH and cooperative electrostatic interactions. This in turn regulates their phase separation propensities, mechanical behavior as well as viscoelastic properties.

The models of crowded environments and of the cellular cytoplasm, in combination with the different physics-based and data-driven methods to investigate crowded environments and protein condensation mechanisms showcased in the previous sections, have been applied to study fundamental biological properties and phenomena, such as protein and small-molecule diffusion, protein–protein and protein–ligand binding, and the viscoelastic and surface properties of condensates inside cells. In this section, we summarize the main mechanistic insights provided by recent simulations about how the physical behavior of biomolecules, protein structure and dynamics and their phase separation behavior are affected by crowding and the cellular environment (Figure 3). Interestingly, in some instances unexpected results are observed, such as increased diffusion rates in the presence of crowding.

### Diffusion

A well-established effect of crowded environments is the reduced diffusion rate of macromolecules and metabolites, attributed to volume exclusion by the crowders, which act as obstacles to free diffusion, as well as to the weak interactions with crowders. Such reduced diffusion rates of proteins or substrates in crowded environments was measured in several studies.^27,59,126^ However, contrary to general expectations, studies of the polymerization of actin and tubulin demonstrated their higher diffusion rates in the presence of crowders, resulting in faster elongation of cytoskeletal filaments compared to more dilute environments.^21,49,50^

Usually, it is observed that volume exclusion prevents the free diffusion of macromolecules^16^ but a few studies have also focused on other effects of volume exclusion. Kompella et al.^65^ observed protein subdiffusion in BD simulations, which was due to the rattling motion of cages formed by crowders that evolved in absence of any significant interactions between the tracer protein, chymotrypsin inhibitor 2, and the crowder protein BSA. In the presence of lysozyme crowders, however, the subdiffusive effect was lost, since there were more protein-crowder interactions. Previous works found contrasting results for the diffusion of molecules in crowded environments. Bellotto et al. investigated the diffusion of proteins inside the *E. coli* cytoplasm using FCS experiments and BD simulations, and observed that the diffusion slowdown depended moderately on the protein size.^127^ Raczyłło et al. investigated the diffusion of metabolites in environments crowded with Ficoll using NMR experiments, BD simulations and Stokesian simulations, and observed that the diffusion slowdown depended on the metabolite size.^66^ This was also demonstrated in a recent MD simulation study of SH3 in the presence of protein crowders like GB1, lysozyme, BSA and ovalbumin, or metabolites like urea and sucrose. The authors reported that while the Stokes–Einstein equation works for systems crowded with metabolites, it does not hold true for protein crowders due to transient clustering of interacting proteins.^91^

The role of protein-crowder interactions on diffusion was demonstrated in recent studies.^23,24^ The authors used PEG and Ficoll as crowders in MD simulations and experiments, and the viral enzyme NS3/4A as a tracer protein. Two types of representation for the crowder PEG were used in this study; the all-atom representation of PEG included the effects of both volume exclusion and weak interactions with the tracer protein, and a CG model of folded PEG, which had no interactions with the tracer protein. The authors observed 40% reduced translational diffusion rates of the enzyme’s peptide substrates in the presence of both PEG models. The rotational diffusion rates were also found to be lower, accompanied by increased formation of transient clusters of tracer protein with PEG as compared to that observed with Ficoll. This was found to be due to the occurrence of more interactions between PEG and the enzyme, and is in line with experimental evidence. The authors also observed that while the affinity of the enzyme for its substrate did not change with the addition of crowders, its activity rate in the presence of the substrate decreased with PEG and increased with Ficoll.

The contribution of weak interactions between target proteins and crowder proteins was further explored using BD simulations and experiments of fluorescence recovery after photobleaching (FRAP) to investigate the diffusion rates of small drug-like molecules.^26^ The authors observed, for one of the small molecules investigated, fluorescein, decreased diffusion rates in the presence of protein crowders such as BSA and lysozyme due to weak interactions between the small molecule and the crowders. Surprisingly, the authors also observed increased diffusion rates for two small molecules, doxorubicin and SB216763, in the presence of protein crowders. The increased diffusion rates were attributed, in the case of SB216763, to reduced self-aggregation of the small molecule due to binding to buried binding sites in the protein crowder BSA and, in the case of doxorubicin, due to the ability of protein crowders in preventing binding of the small molecule to the glass surface.

The diversity of crowders in the system (also referred to as polydispersity in the literature) has also been known to affect the diffusion rates of tracer molecules, as discussed by Miyaguchi.^128^ The effects of polydispersity in crowded environments is discussed in detail in previous reviews.^19,129^ The importance of heterogeneity of crowders to account for all types of transient interactions was demonstrated in a combined experimental study and stochastic particle simulations of nanoparticles inside cells by Garner et al.^70^ By varying the cytoplasmic viscosity in the simulations, the authors were able to reproduce the 10-fold range of differences in diffusivity of the nanoparticles observed experimentally between individual cells. They claimed that the cytoplasm is a heterogeneous environment with subcellular components that have a range of effective viscosities, which contribute to the effects seen in experiments. The effect of softness of crowders as well the shape of tracer proteins has been explored in another study, where the authors used a modified potential to calculate the interactions between soft or flexible crowders and hard or compact crowders by implementing low resolution simulations (CG MD or BD).^68^ They found that softer crowders do not reduce the diffusion rates significantly. The study further revealed that if the shape of the tracer protein is cylindrical, instead of a sphere, it may diffuse similarly in the presence of soft or hard crowders, and may even diffuse faster in the presence of hard crowders.

In addition to volume exclusion and weak interactions with crowders, confinement can also modify diffusion rates. Śmigielet al. used BD simulations to elucidate the effect of confinement imposed by cell membranes, which resulted in variations in protein diffusion rates observed in their single-molecule displacement mapping experiments.^58^ In their simulations, the particles were reflected off of the boundary instead of maintaining periodicity, and they found that the diffusion rate was faster in the center and decreased at the poles of the model, which was consistent with their experimental observations. This could be attributed to the reflections from the boundary, but also to accumulation of damaged proteins in the poles. Protein location inside cells is another aspect that could be explored and incorporated into models and simulations of cellular environments.

### Protein Association

Protein binding to a partner (protein, nucleic acid or small molecule) inside cells is a crucial step for several processes. While association rate constants for protein-target binding are expected to be lower in comparison to *in vitro* conditions due to slower diffusion rates, the diverse effects of the cellular environment over protein-target binding are still largely unknown.^130^

Previous works investigated the effect of crowding over kinetic rates and paths for protein–ligand binding. Recently, the binding between trypsin and the small molecule benzamidine was studied across a wide range of crowder concentrations using MD simulations and Markov state modeling.^90^ As compared to dilute solutions, the association rate constant (k_*on*_) was 15–20 lower in crowded environments. Initially, the k_*on*_ values increased as the crowder concentration was increased, but when the fraction of crowders went beyond 15% of the volume, the k_*on*_ values decreased. According to the authors, this was because at high crowder concentrations, the ligand was trapped in one of the key intermediate states for binding, where the hindrance posed by the crowders limited the diffusion of the ligand to its target binding site. In another study, the impact of crowding on the binding of the pyrazolopyrimidine (PP1) ligand to the Src kinase protein in the presence of high concentrations of BSA protein crowders was investigated.^27^ The authors observed reduced ligand binding efficacy as the BSA concentration was increased, because of weak attractive interactions between crowders and the ligand. Interestingly, the simulations suggest that there is a change in the protein–ligand binding pathway, in comparison to the dilute solution. However, more binding events are required to confirm whether this change in binding paths in the presence of crowders is statistically significant. In MD simulations of the SARS-COV main protein and its inhibitory drug, along with 8 GB1 crowder proteins and 10 cytoplasmic metabolites, the inhibitor was unable to bind to its target protein due to multiple interactions with the metabolites.^131^ In their subsequent publication, the authors demonstrated how metabolites, such as UD1 and ATP, may also form clusters while interacting with the tracer proteins and ligands of the system.^92^ This study further demonstrates the necessity to include metabolites in the cytoplasmic models to replicate in vivo interactions in molecular simulation studies.

Several independent studies investigated the effect of crowding over protein–ligand binding in simpler model systems. Majumdar and Mondal used a model of a cavity-ligand system with C60 fulerene as crowder.^132^ They found that the crowders facilitated ligand binding by reducing the free energy barrier by volume exclusion. This effect also facilitated protein desolvation. A target search model for ligand-active site binding considered the reversible binding-unbinding reactions with the Brownian motion of particles, and the simulations showed a higher chance of binding due to multiple chances for discovery of the interaction sites.^71^ In another study, the effect of crowding on the allosteric cooperativity of divalent biomolecules binding to monovalent molecules was studied. Using MC, BD and CG MD simulations, the authors observed that divalent binding occurs only in the presence of bulk crowders when the divalent molecules outnumber the monovalent molecules associated with them.^136^

The effect of lysozyme crowders on the association of the side-by-side dimer and the domain-swapped dimer of the B1 immunoglobulin-binding domain of protein G was studied by Pradhan et al.^56^ The CG MD simulations revealed that the lysozyme crowders destabilize native contact formation in both types of dimer due to the attractive interactions between crowder and the protein, which overcomes the excluded volume effect. This result was in contrast to what the same authors observed in BD simulations with the particle-based reaction-diffusion model, with only LJ potentials applied on the spherical tracer and crowder proteins. Here, stabilization of dimers was seen due to attractive LJ interactions that hold the monomers together and improve the chance of dimerization. In qualitative agreement with experiments, they observed that the domain swapped dimer is more likely to form in both diluted and crowded conditions.

The association of DNA binding proteins to their target DNA was studied in an MC model by Punia et al, where the time of target search and its pathway were characterized based on the location of the target relative to the protein and protein-crowder affinities. When the association rate constant of proteins with crowders (c_*on*_) was greater than with the DNA (k_*on*_), the search time reduced as compared to dilute solutions and when c_*on*_ = 0. They concluded that instead of a random walk, the crowder-assisted search pathway helped the protein to access the target DNA sites blocked by crowders via a new channel, thus allowing it to efficiently scan and recognize its target site.^50^

*In-silico* studies of cytoskeleton polymerization explored the effects of cytoplasmic proteins on the rate of cytoskeletal fiber association. Recently, MC simulations of microtubules in the presence of low and high molecular weight crowders were used to study the collective physical effects of crowding on the growth of cytoskeletal polymers inside cells. The simulations provided evidence that the diffusion of elements and elongation rates of filaments in the presence of smaller crowders is reduced due to an increase in microviscosity, while it is not affected by larger crowders. The *denovo* nucleation rate however increased due to crowding at critical concentration in a size independent manner according to the polymerization kinetics experiments performed. The combination of these effects thus accounted for the bulk elongation rates measured in experiments.^48^ In a similar study by Wang et al, the experimental dissociation constant (K_*d*_) for the dimerization of wild type and dimer-only mutant of prokaryotic tubulin proteins BtubA and B was reproduced in MC simulations. Their study indicated that the volume exclusion from the crowded environment *in vitro* played a more important role than the weak quinary interactions toward the decreasing K_*d*_ of BtubA and B. This effect led to the facilitation of the polymerization process even if the local quinary interactions were modified. However, this trend changed at higher temperatures, where the enthalpic effects reduced the entropy of the system and promoted dimerization instead.^49^ Short MD simulation of 5 ns also revealed a similar trend of polymerization in crowded conditions.^21^

### Protein Stability and Folding

Maintaining the stability of proteins is crucial for their proper functioning, and a key focus lies in understanding the equilibrium between the stabilizing effects caused by volume exclusion in a crowded environment, and the potentially destabilizing effects of weak interactions, which can differ among protein crowders.

The effect of charges on the stability and folding of superoxide dismutase (SOD1) was investigated in a self-crowded environment.^72^ The native conformations of SOD1 with screened charges (−1 e), the uncharged native protein or its variant, with a single point mutation (G41D), that adds a negative charge (−2 e), were used as model systems. The authors found a folding intermediate state sensitive to solvent ionic strength, that may play an important role in the folding pathway. The addition of charge led to an increase in interprotein interactions, and in the mutated SOD1 it led to aggregation, which could be responsible for amyotrophic lateral sclerosis (ALS) disease. However, in the absence of charged crowders, inert or less interacting crowders like fullerenes promoted more intraprotein contacts, which promoted the globular metastable states of α-synuclein and protein-G.^25,78^ In the human cytoplasmic models, the hinge-bending landscape of human phosphoglycerate kinase (PGK) was simulated. The simulations suggested that the crowded environment promotes the stability of semiopen hinge-bending states of PGK.^47^

The protective effect of crowded environments over proteins upon temperature increase has also been investigated in different studies. Timr at al reported that high temperatures affect the protein crowders BSA and lysozyme, which may lead to their unfolding along with the test protein, chymotrypsin inhibitor 2 (CI2). This in turn resulted in decreased volume exclusion effect and a destabilization of the test protein. They suggested there is a crossover temperature beyond which crowding does not provide resistance to changes in temperature, leading to unfolding at higher temperatures.^77^ To avoid the unfolding of crowders, Katava et al. tested the stability of lysozyme crowded with glycerol, where only one protein is affected by the gradual increase in temperature and all other molecules were in frozen states using REST2 MD simulations.^82^ Here, lysozyme experienced 60% volume exclusion when present in a powder like state. The authors showed that the effect of volume exclusion can be overcome if the proteins in the environment are sensitive to increases in temperature or if there are electrostatic interactions that take effect in the crowded environment.

### Material Properties of Phase Separated Condensates

Mesoscopic material properties of biomolecular condensates, such as viscosity, viscoelasticity, and surface tension, play a crucial role in dictating many cellular functions. Consequently, a large number of computational studies have been performed with the goal to understand and estimate these material properties and their connection to the functional behavior of condensates. To decipher the relationship between amino acid sequence and the material properties of charged IDPs, Mittal and colleagues^100^ have recently explored how alterations in charge patterning within IDP sequences affect the diffusion coefficient, viscosity, and surface tension of the condensates. They used two types of sequences for this study; model proteins consisting of negatively charged glutamic acid (E) and positively charged lysine (K) residues and two different naturally occurring charge-rich proteins, LAF1’s RGG domain and the DDX4’s N-terminal domain. The analyses of the material properties showed that charge patterning resulted in monotonic changes in them, despite the diverse sequence compositions of the model proteins and naturally occurring proteins. With increasing charge segregation, the diffusion coefficient of protein chains within the dense phase of the condensate decreased, while the dense phase viscosity and surface tension at the condensate-water interface increased systematically. What is interesting here is that the rate of change in these material properties with varying charge distribution was found to be nearly identical between model and natural proteins, underscoring the interdependence of these properties across a wide range of sequence compositions. These observations emphasize that sequence charge patterning can modulate the material properties both within the dense phase and at the interface of charge-rich IDP condensates, without the need to change external conditions such as temperature and salt concentration.

One of the most crucial material properties of condensates is their surface tension, which stems from the interactions among the constituent molecules and their interactions with the solvent. Surface tension of condensates is known to significantly influence the equilibrium properties of condensates, by controlling both their morphology and internal organization. Using the well-known sticker-spacer model of associative polymers, which closely represents one-to-one interactions in biological systems like the algal pyrenoid (composed of the enzyme Rubisco and the linker protein EPYC1), Wingreen and colleagues^133^ established a strong correlation between polymer sequence and the surface tension of condensates, and consequently with the thermodynamic critical temperature *T*_*C*_ associated with the polymer solution.

The transition of condensate phase from liquid-like to solid/gel form has been known to be closely related to the onset of multiple neurodegenerative disorders, and can potentially be characterized by their change in viscoelastic properties. In order to decipher the molecular mechanisms underlying the changes in viscosity associated with liquid to solid transition of condensates, Espinosa and co-workers^134^ tested the performance of several powerful computational methods which are rooted in the concepts of polymer physics. They applied these techniques to determine the droplet viscosity of a set of 7 different IDPs and 5 peptide/RNA complex coacervates using a sequence-dependent CG model. They found that the viscosity of the phase separated condensates in each case rises with increasing chain length of the proteins or RNA and therefore with increasing molecular mass. They also estimated the correlation between viscosity and the sequence composition across the different studied IDPs and found that viscosity is proportional to the abundance of amino acids that act as binding sites for associative interactions, which are popularly known as *stickers* in the protein phase separation framework.

## Perspectives and Conclusion

Taken together, the studies reviewed here provide a snapshot of the state-of-the-art of methods to model and simulate macromolecular crowding, cellular environments and biomolecular condensates. Till date, simulation methods are being actively developed to model the cellular cytoplasm (such as CELLPACKgpu^42^ and python scripts to build *E. coli* cytoplasm models^135^), and to simulate and analyze large and heterogeneous systems (such as the GENESIS software package^73,75,76^ and the set of analysis tools SPANA^89^). Remarkably, models of complete bacterial cells are available at this point of time,^37,41^ including the membrane and macromolecules in the cytoplasm. The next challenge now is to build the tools that enable the simulation of such large models.

Interestingly, the mechanistic insights provided by simulations can sometimes differ from expectations (such as increased diffusion rates, instead of slower diffusion rates, for small molecules in the presence of crowding^26^). This showcases the importance of a broader investigation of the effects of crowding over biological phenomena. Such studies can not only provide unexpected information, but will also pave the way for the formulation of general rules for the effects of crowding over protein folding and ligand binding. For instance, are the kinetic rates, pathways and intermediate states for protein folding and ligand binding the same in crowded environments and *in vitro*? While some of the studies highlighted in this review already identified differences in ligand binding in crowded environments, in comparison to *in vitro* conditions, more simulations are required to understand how widespread the effects of crowding are, and experiments are required to validate the results from simulations. Moreover, knowledge about the diffusion rates of drugs and kinetic rates for protein-drug binding inside cells may have important applications in drug design, as previously discussed in Kasahara et al.^27^ For instance, drugs can be modified to have less weak interactions with protein crowders, displaying faster diffusion rates and faster association rate constants upon binding to their target proteins in vivo, leading to higher drug efficacy. The same holds true for biomolecular condensates, as their dysfunction is closely associated with several diseased states, including neurodegeneration, cancer, viral infections and cardiac disease. Deciphering the molecular mechanism of condensate formation as well as estimating their mechanical behavior in a closely replicated cellular environment could pave the way for developing new therapeutic targets for these conditions. Similarly, simulation set-ups for underpinning condensate diffusivity and visocelastic behavior in both liquid-like and dense or solid-like state in a closely matched cellular environment which is closely linked to condensate driven neurodegenrative disorders can lead to the identification or design of condensate modifying therapeutics. A step forward toward this effort could be taken by estimating diffusive behavior of model condensates in the presence of simple, nonbiological crowders with effective interaction, as discussed in previous sections. There is also the need to understand multicomponent phase behavior of condensates and how the presence of cellular material in the cytoplasmic state can facilitate their phase separation process. While physics-driven methods are currently being implemented to accurately simulate systems with more than a single component, the rise of machine learning models offers tremendous potential for predicting the components of biomolecular condensates in cells and uncovering previously unknown components of such systems, provided sufficient experimental data is available for model training.

In the next few years, we anticipate further method development and more studies investigating condensate formation and the effects of macromolecular crowding and cellular environments over protein-target binding, diffusion rates of macromolecules and small drug-like molecules, and protein stability. However, the success of these studies depends on the availability of data (such as diffusion rates of molecules, or rate constants for protein-target binding) from experiments performed inside cells to benchmark the methods and validate simulations. Experimental methods such as FRAP, to measure diffusion rates, and NMR, to detect protein conformational changes, can be used to investigate proteins inside cells, providing useful benchmarks for the effects of crowded environments.

## Data Availability

All information and data is included in the text, and no new data or software were used in the preparation of this manuscript.
